# Supplementary material for: Integration of daytime radiative cooling and solar heating for year-round energy saving in buildings
Source: Nat Commun. 2020 Nov 30;11:6101. doi: 10.1038/s41467-020-19790-x (PMC7705009; doi:10.1038/s41467-020-19790-x)
Supplement: Supplementary file 1 — Supplementary information [file 41467_2020_19790_MOESM1_ESM.docx]

**Supplementary material to:**

Integration of daytime radiative cooling and solar heating for year-round energy saving in buildings

Li et al.


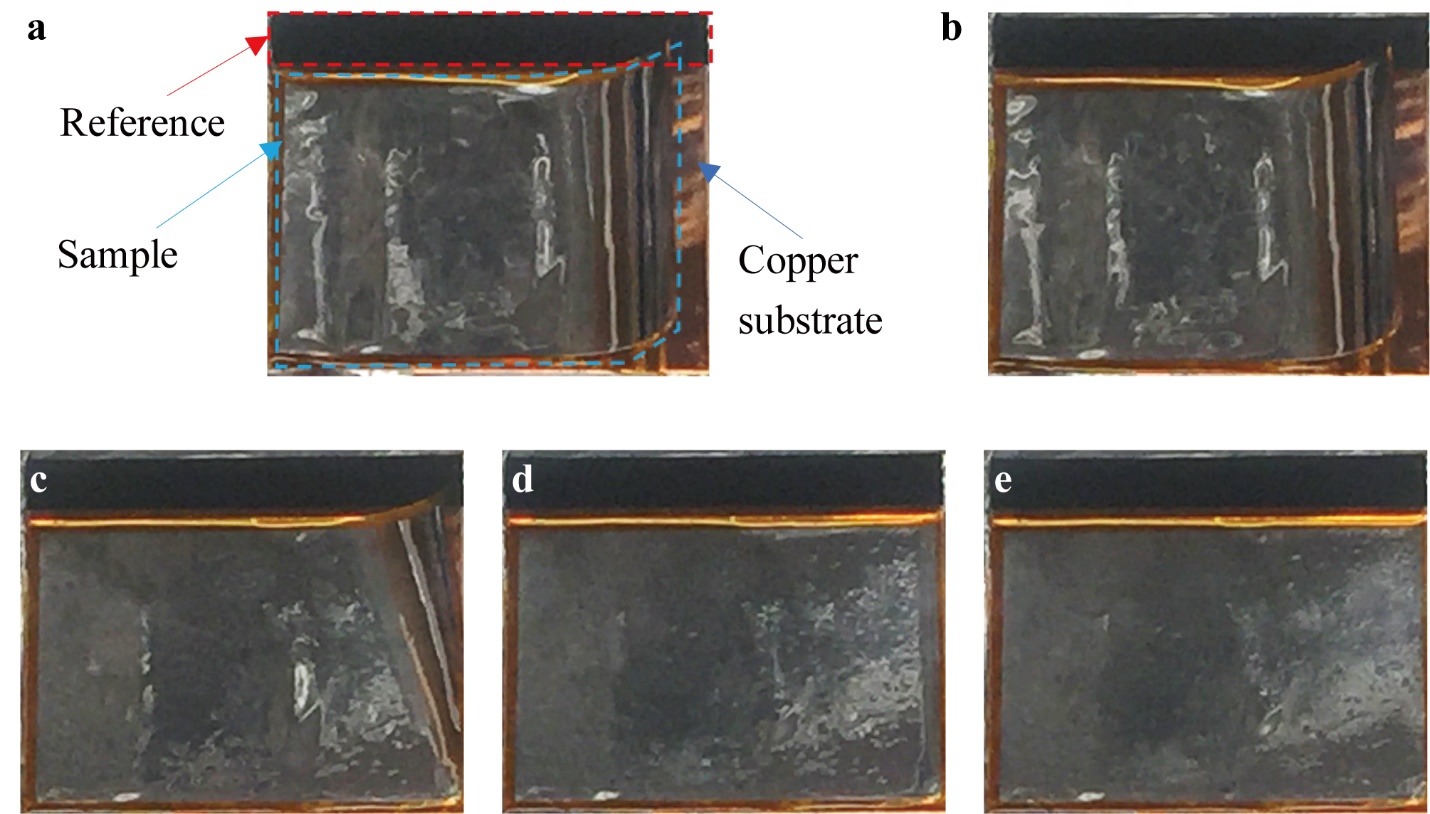


**Supplementary Figure 1**. Photos of the cooling material on the copper plate after applying different voltage. **a**, 0 kv; **b**, 0.5 kV; **c**, 1 kV; **d**, 2 kV; **e**, 3 kV.


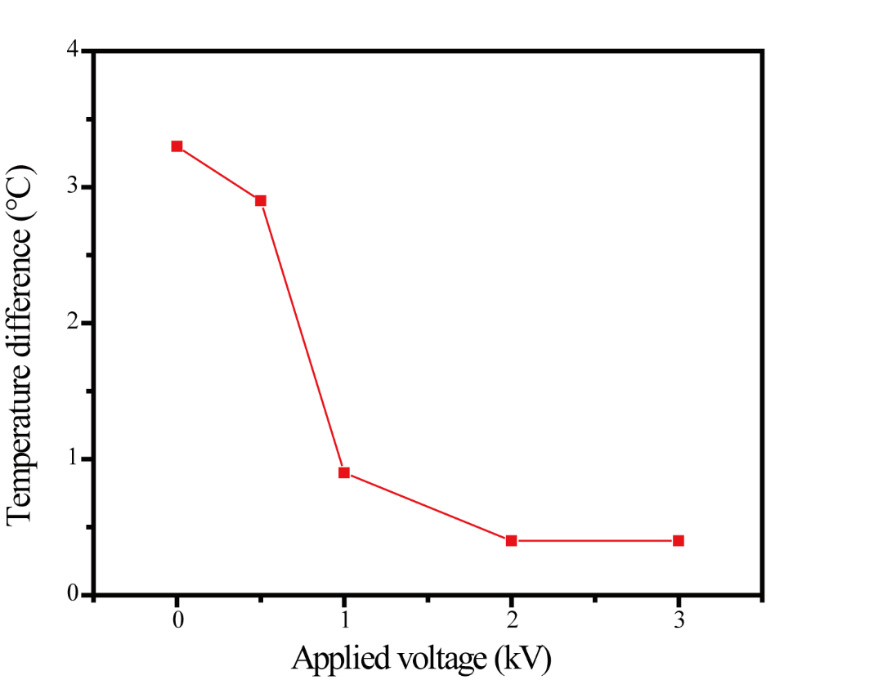


**Supplementary Figure 2**. Average temperature difference between the reference and the sample over applied voltage.

**
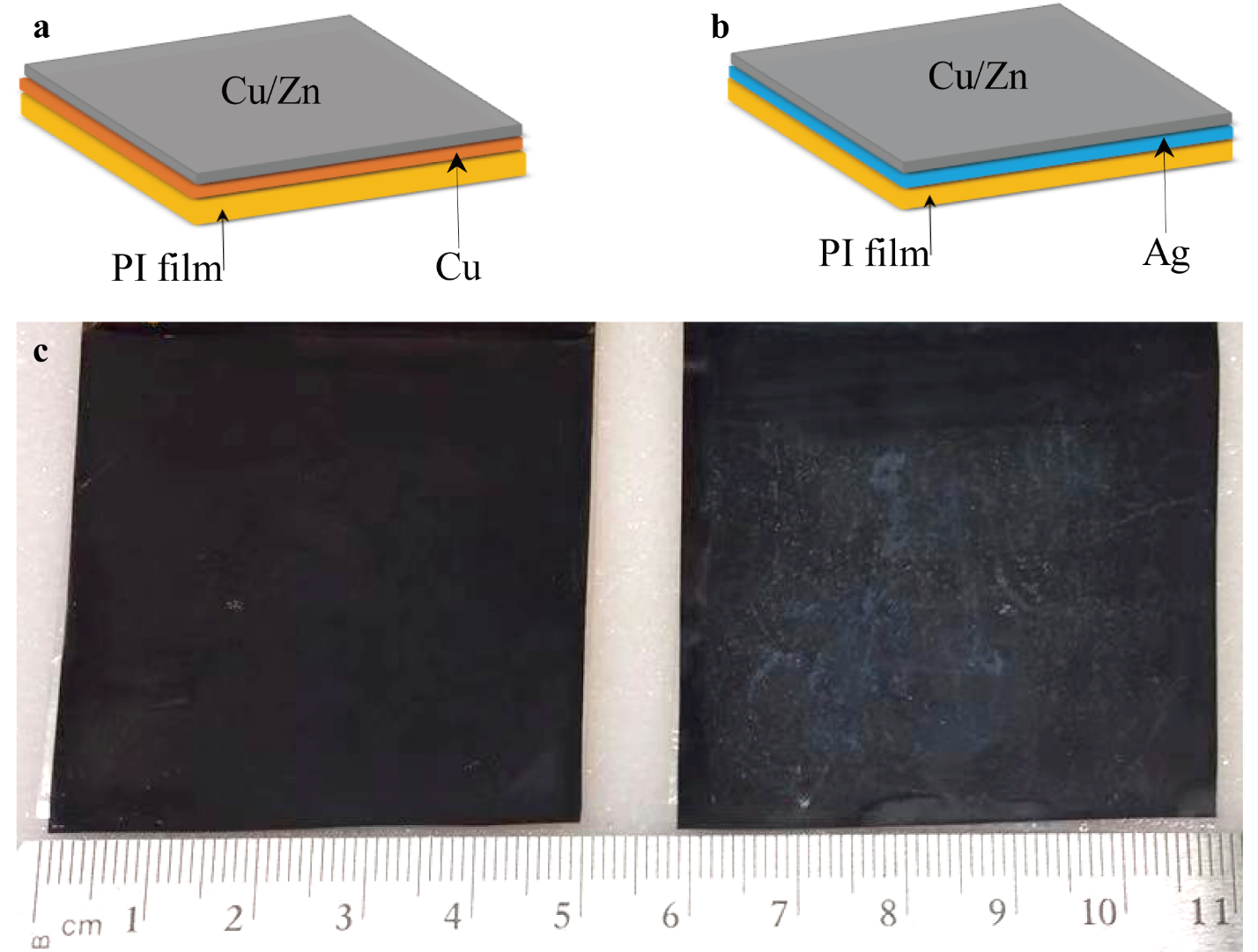
**

**Supplementary Figure 3**. **Optimized experiment of heat materials**. **a**, Heating material with copper layer, and corresponding optical image in (**c**, Left). **b**, Heating material with silver layer, and corresponding optical image in (**c**, right).


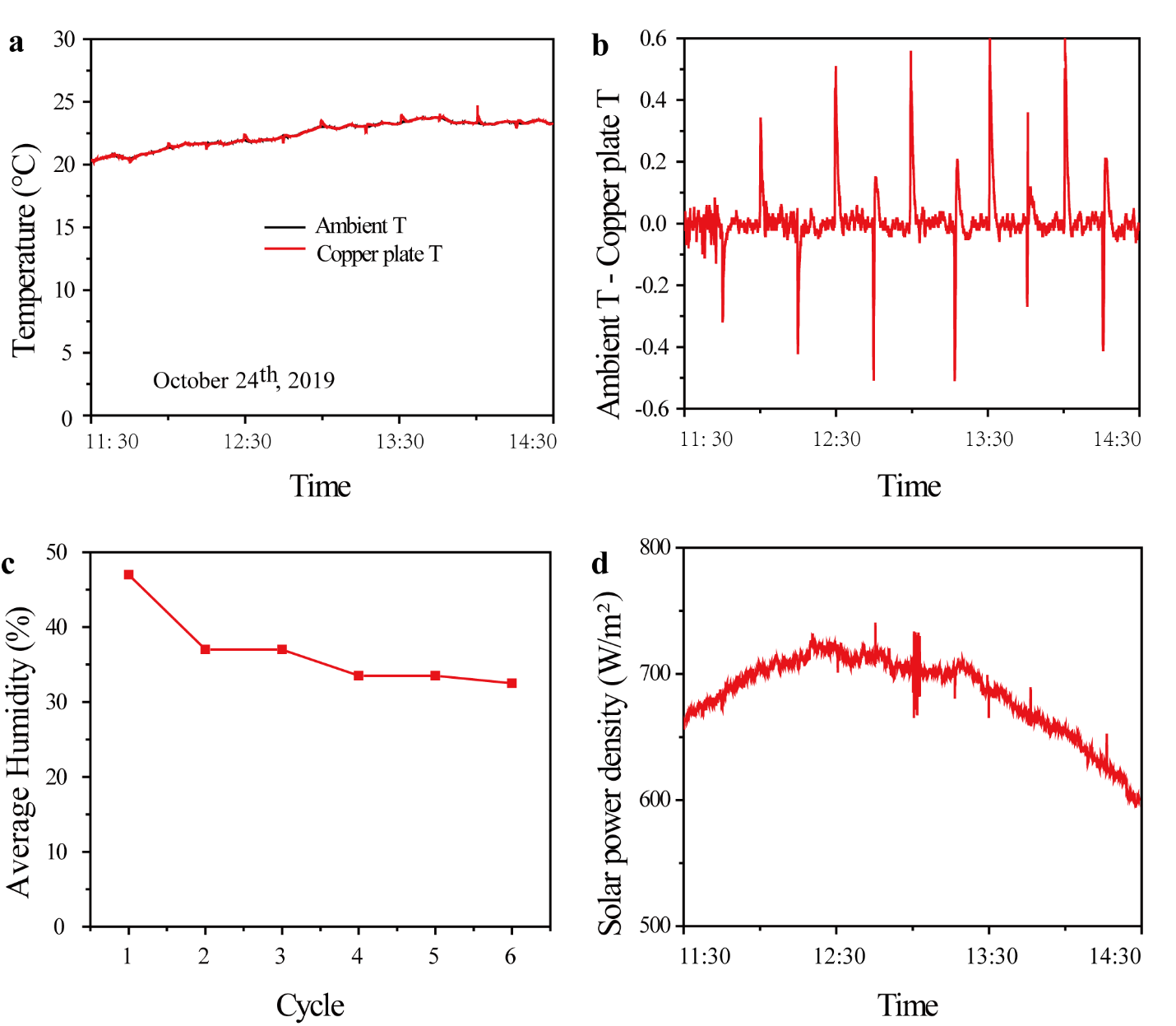


**Supplementary Figure 4**. **Testing equipment and weather parameters of outdoor experiment**. **a**, Ambient temperature and copper plate temperature over time. **b**, The difference between ambient and copper plate temperature over time. **c**, Humidity over time. **d**, Solar power density during the testing period from 11:30 to 14:30 on October 24^th^, 2019 at Duke University, Durham, NC.

**
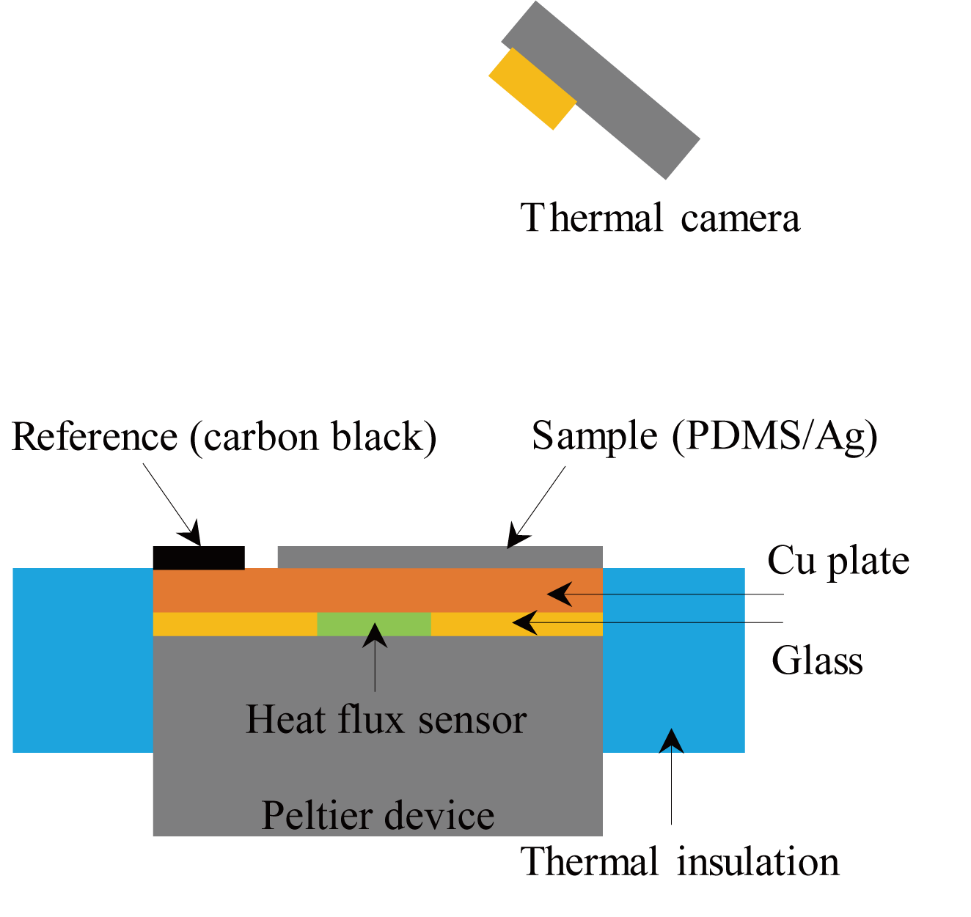
**

**Supplementary Figure 5**. Schematic of the testing system of thermal contact conductance.

**
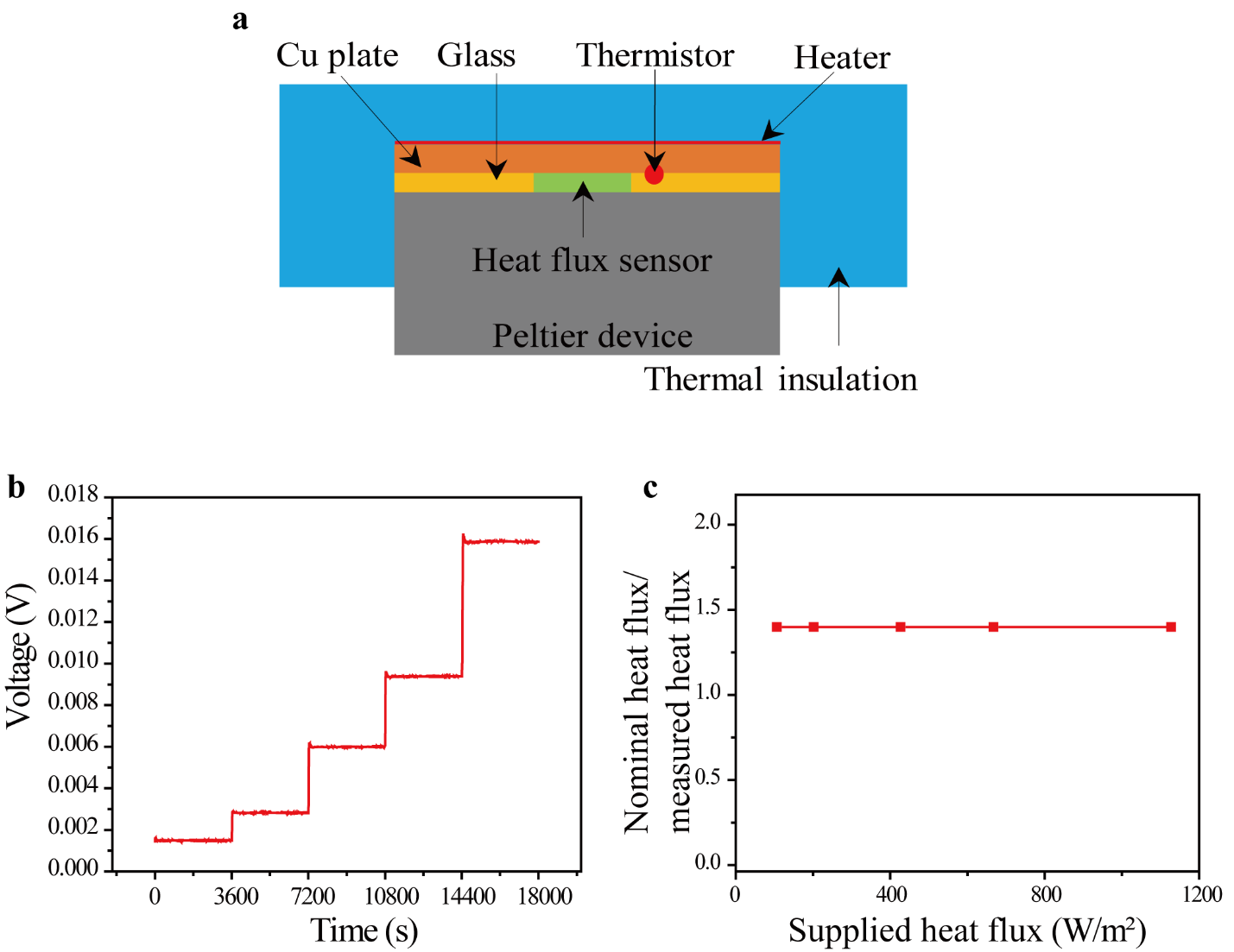
**

**Supplementary Figure 6**. **Calibration of the testing apparatus**. **a**, Schematic of calibration of the testing system. **b**, Voltage reading of heat flux sensor over time when applying different power density (106.68, 201.79, 427.18, 667.38, 1127.72 W/m^2^). **c**, The ratio of nominal heat flux to measured heat flux over the supplied heat flux.

**Supplementary Table 1**. Summary and comparison of the synergistic technologies based on solar heating and radiative cooling reported in literature.

|  | Daytime sub-ambient cooling | Solar heating  with selective absorber | Heating performance  Absorption (A) Emissivity (E)  Transmittance (T) | Cooling performance  Reflectance (R)  Emissivity (E) | Tuning method |
| --- | --- | --- | --- | --- | --- |
| Hu et al.^1^ | × | × | 300-2000 nm  A = ~ 90%;  8-13 µm  E = ~ 80% | 300-2000 nm  A = ~ 90%;  8-13 µm  E = ~ 80% | — |
| Vall et al.^2^ | × | √ | Commercial Ti absorber | Black paint | (Hypothetical) Mechanical |
| Hu et al.^3^ | × | × | — | — | Tandem approach |
| Ono et al.^4^ | √ | × | 8-13 µm  E = 5.4% | 8-13 µm  E = 63.6% | Thermal |
| Mandal et al.^5^ | × | × | 300-2000 nm  A = ~ 90%;  4-18 µm  E = ~ 90% | 300-2000 nm  R = ~88%;  8-13 µm  E = ~60% | Electrical |
| Mandal et al.^6^ | √ | × | 300-2000 nm  T= ~ 94%;  4-18 µm  E > 90% (Water) | 300-2000 nm  R = ~95%;  8-13 µm | Mechanical |
| Zhao et al.^7^ | √ | × | 300-2000 nm  A = ~ 95%;  4-18 µm  E = ~ 94%  (PDMS) | 300-2000 nm  R = ~93%;  8-13 µm  E = ~94% | Mechanical |
| Our work | √ | √ | 300-2000 nm  A = 93.4%;  4-18 µm  E = 14.2% | 300-2000 nm  R = 97.3%;  8-13 µm  E = 94.1% | Mechanical |

**Supplementary Note 1**. Reliability issues

1. Analysis of the potential impact of dirt

It is expected that the interfacial thermal resistance could be impacted when dirt gets into the interface between the PI film and the substrate. When manufacturing the proposed dual-mode heating and cooling device, it is critical to package the film, the underneath substrate, and the rolling components into a tightly sealed modular system to highly reduce the dirt penetration risk. In addition, operation maintenance architectures such as brush cleaners installed at the separation edge of the heating/cooling film will intermittently clean the surface of the underneath substrate and help remove dirt that may appear between PI film and substrate.

2. Analysis of the potential impact of humidity

As shown Supplementary Fig. 7, the effect of humidity on interfacial thermal resistance is also demonstrated. The result shows that the good thermal contact can maintain for two days when the relative humidity is in the range of 40% to 60%. Even in the rare case of > 95% humidity, a good thermal contact still can last for more than one day. In practical applications, periodic “charging” will be performed to ensure continuous good thermal contact.


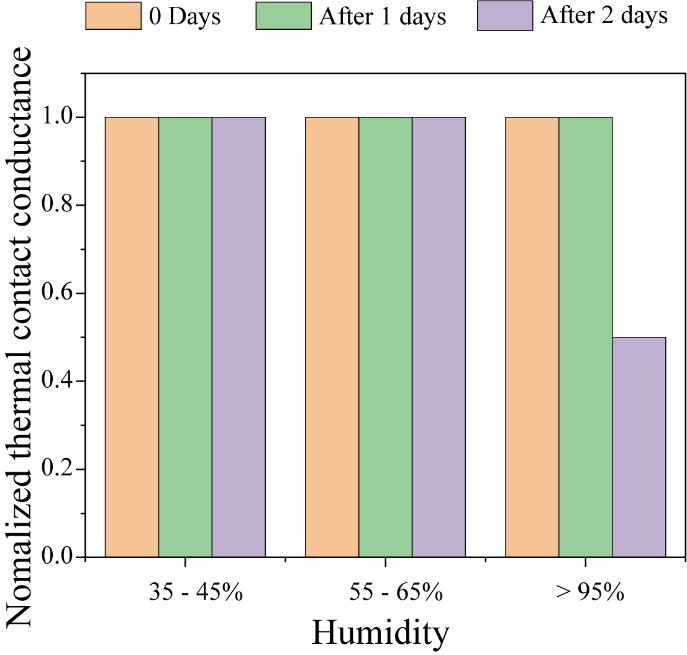


**Supplementary Figure 7**. The thermal contact over time at different humidity levels

**Supplementary Note 2**. Heating material

For heating material, on the copper film, a layer of zinc film of 1 µm thick was electrodeposited (voltage: 2 V, anode: zinc metal, electrolyte: 0.25 M ZnSO_4(aq)_), followed by galvanic replacement reaction with 0.12 mM CuSO_4(aq)_, and the heating material was obtained after deionized water washing and drying. As shown in Supplementary Fig. 8, it can be found with the increase of reaction time with CuSO_4(aq)_, the absorption of both 300 - 2000 nm and 4 - 18 µm increased. The observations can be attributed to the size of copper/copper oxide clusters is increased (as shown in Supplementary Fig. 9). Specifically, the absorption of 300 - 2000 nm stems from the localized surface plasmon resonances of the Cu nanoparticles. The wide size distribution of the Cu nanoparticle clusters results in broadband absorption, which is beneficial for solar heating. As the reaction time increases, both near-field coupling and the total volume of light–matter interactions increase, which promotes broadband absorption in the solar spectrum. For 4 - 18 µm part, the nanoparticle layer behaves as a lossy effective medium because of the small cluster size compared to thermal radiation wavelength. Therefore, longer reaction time leads to a higher attenuation of light in both solar and mid-IR regimes^8^.


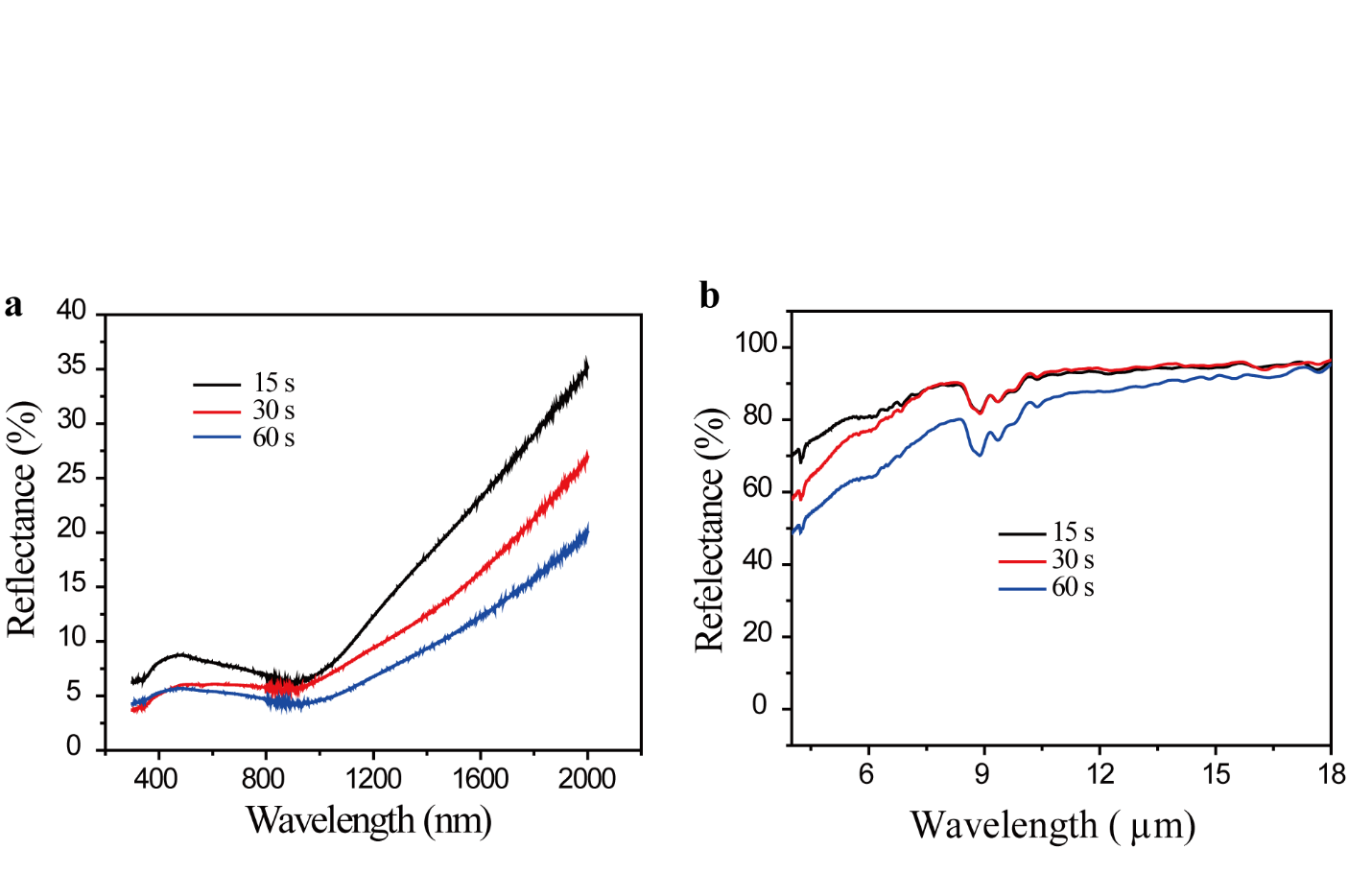


**Supplementary Figure 8**. **Optical properties of heating materials**. **a** and **b**, Reflectance spectra of heating materials of different reaction time with CuSO_4_.


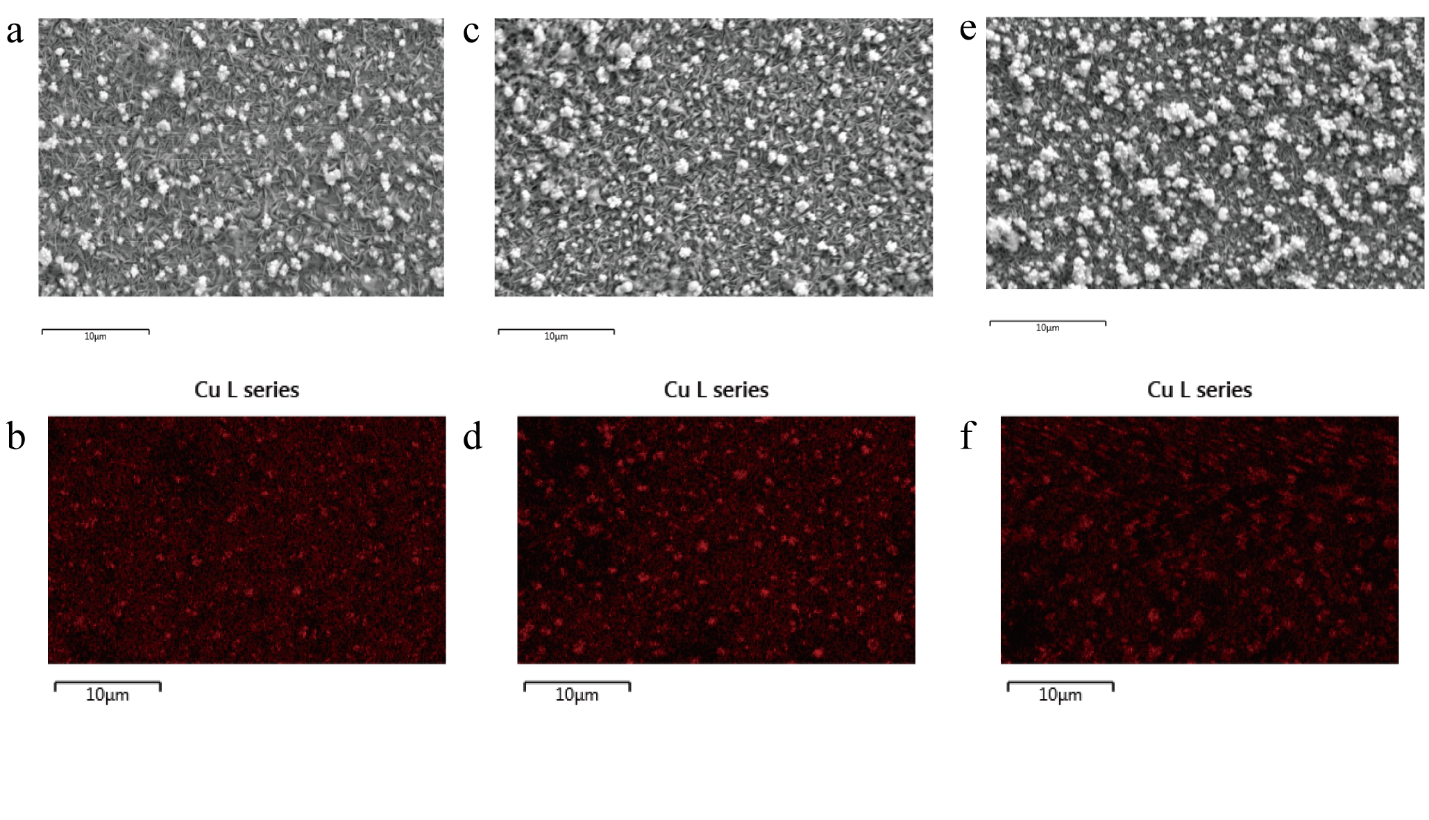


**Supplementary Figure 9**. **SEM and Cu element mapping images of the heating materials**. **a and b**. reaction time of 15 s. **c and d**. reaction time of 30 s. **e and f**. reaction time of 60 s.

**Supplementary Note 3**. Numerical models for radiative cooling and solar heating

Material cooling power:

Planck’s law describes the spectral radiance of a black body for wavelength ($\lambda$) at absolute temperature *T* is given by:

$$I_{BB}\left( T,\lambda\right)=\frac{2hc^{2}}{\lambda^{5}}\frac{1}{e^{\frac{hc}{\lambda kT}}-1} (1)$$

where *c* is the speed of light, $h$ is Plank constant and *k* is the Boltzmann constant. The radiative cooling power density of surface film can be obtained by integrating the spectral radiance density over atmosphere long-wave infrared radiation transmission windows (LWIR), 7-13 um：

$p_{rad}\left( T \right)=2\pi\int_{0}^{\frac{\pi}{2}} \int_{7 um}^{13 um} I_{BB}\left( T,\lambda\right)\epsilon_{film}\left( \lambda,\theta\right) sin\theta cos\theta d\lambda d\theta\left( 2 \right)$ $\epsilon_{film}\left( \lambda,\theta\right)$ is the spectral emissivity (absorptivity) of the dual-mode surface film. Specifically, $\epsilon_{film}$= 0.941 for cooling mode and $\epsilon_{film}$=0.142 for heating mode and are assumed to be angle-independent. Correspondingly, the power density of absorbed thermal radiation from the atmosphere is given by:

$p_{amb}\left( T_{amb} \right)=2\pi\int_{0}^{\frac{\pi}{2}} \int_{7 um}^{13 um} I_{BB}\left( T,\lambda\right)\epsilon_{film}\left( \lambda,\theta\right)\epsilon_{atm}\left( \lambda,\theta,H_{2}O \right) sin\theta cos\theta d\lambda d\theta\left( 3 \right)$

where, $T_{amb}$ is the ambient temperature and $\epsilon_{atm}\left( \lambda,\theta,H_{2}O \right)$ is the atmospheric emissivity. The atmospheric emissivity $\epsilon_{atm}\left( \lambda,\theta,H_{2}O \right)$ at LWIR (7-13 um) was calculated using ATRAN - a web-based software provided by SOFIA Science Center^9^. The cooling power density (W/m^2^), *p*_cool_, after taking the solar heat gain and the convective/conductive loss into consideration, is given:

*p_cool_* = *p_rad_* – *p_amb_* - *h_L_*∆*T* – *Iβ* (4)

where *h_L_* is heat transfer coefficient of convective and conductive loss, Δ*T* is the temperature difference between cooling material surface and environment. *I* is the global horizontal solar radiation. *β* is sunlight absorption coefficient of cooling film. The model value of Fig. 4 is calculated by this method.

System cooling-mode energy saving:

Potentially, there are quite many application methods for the proposed dual-mode radiation heating and radiative cooling materials in buildings. We demonstrate an example application of integrating the material with building envelopes to provide space heating and cooling energy using heat exchangers. At this system level application, a comprehensive integrated analysis of the proposed device and the subject building is needed, which creates hourly performance simulation for the 16 cities throughout a whole year. In addition, in order to evaluate the energy savings of the dual-mode device under real application condition, the typical meteorological year (TMY3) ^10^ weather data are used, and the impacts of the humidity and the clouds on cooling capability are evaluated. Therefore, the following calculation algorithms (eq.5 – eq.10) are selected to estimate the cooling power with effective atmospheric emissivity (ɛ_atm_):

*P*_cooling power_ = *P’*_rad_ – *P’*_atm_ (5)

*P’_rad_* = *Aɛ_film_σT_film_*^4^ (6)

*P’*_atm_ = *Aɛ_film_ɛ_atm_σT_amb_*^4^ (7)

where *ɛ_film_ and T_film_* are the emissivity and surface temperature of the film, and *A* is the area*.* The *ɛ_atm_* is given by^11^,

*ɛ_atm_* = *ɛ_atm,c_* (1-0.78CF) + 0.38CF^0.95^ *RH*^0.17^ (8)

*ɛ_atm,c_* = 0.618 + 0.056 $\sqrt{P_{w}}$ (9)

*P_w_* = *P*_0_ exp [(*c_T_T_d_*) / (*T_d_* + *T_0_*)] (10)

Where *ɛ_atm,c_* is the effective sky emissivity under clear skies, CF is the cloud fraction, *RH* is the ambient relative humidity, and *P_w_* is the ambient water vapor partial pressure, *T_d_* is the dew point, *P_0_* = 610.94 Pa, c_T_ = 17.625, and *T_0_* = 243.04 ℃. The hourly values of these weather-related parameters can be obtained from TMY3 weather data.

With heat transfer medium such as water flowing in the heat/cold exchangers or collectors, the cold water will possess varied temperatures with environmental weather changes. As Ref 6 shown, lower temperature water can be directly used for space cooling through indoor systems such as radiant cooling ceilings, which commonly adopts a fluid temperature of 13-18 ℃^12^ in order to avoid surface condensation. In contrast, when the water temperature is higher than this range, the radiative cooling cold water can be supplied to air conditioner side and cool the condenser side to achieve higher efficiency (details seen in the next section). In other words, the cooling power is to directly cool the building spaces when the temperature is below 18 ℃, and to cool the air conditioner condenser when the temperature is above 18 ℃. Therefore, when the temperature is below 18 ℃, that particular hour’s *P_cooling saving_* is calculated by,

*P_cooling saving_* = *MIN* (*P_cooling_*, *P_cooling_ _load_*) (11)

System cooling power analysis for air-conditioner unit

We choose to model the energy saving by considering the case of retrofitting a traditional air-cooled vapor-compression air-conditioner unit with the radiative cooling device using water as the heat transfer fluid, as shown in Supplementary Fig. 10a. The thermodynamic cycles are shown in Supplementary Fig. 10b. The blue line represents the traditional air-cooled AC and the red line represents the AC coupled with radiatively-cooled, below-ambient-temperature water. Points 1-4 represent saturated vapor at low pressure, compressed vapor, saturated liquid at high pressure, liquid after expansion valve. The radiative cooling system provides additional cooling and reduces the condenser temperature, and the new thermodynamic cycle follows points 1’-4’, which is more efficient than 1-4. By comparing the coefficient of performance (COP) before and after installing the radiative cooler, the energy saving can be calculated.


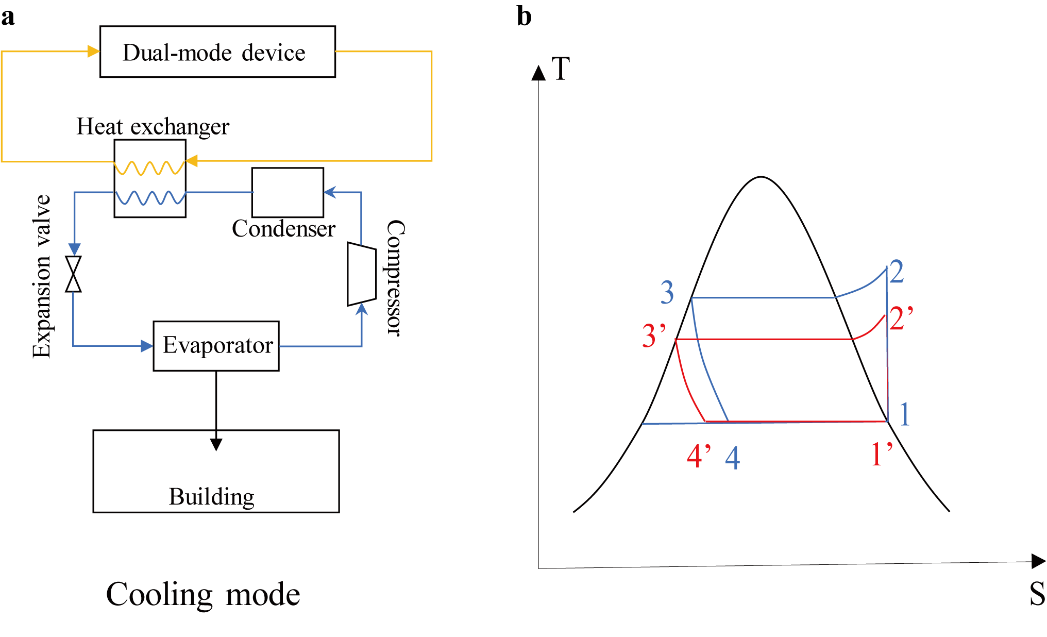


**Supplementary Figure 10**. **Modeling cooling system-level energy savings**. **a**, Scheme for air-cooled AC coupled with radiative-cooling fluid panels. **b**, Thermodynamic cycle diagram of the air-cooled AC with (red) and without (blue) radiative cooling.

For the air-cooled vapor-compression AC, the basic thermodynamic equations (eq.12 - eq.17) are listed below to demonstrate its performance during the “compression-condensation-expansion-evaporation” loop. *P*_load_ is the heat removed from the building for maintaining the comfortable room temperature (22°C) per unit time, which is calculated from *EnergyPlus*. d*m*/d*t* is the mass flow rate of refrigerant. h_1_, h_2_, h_3_, h_4_ are the enthalpies for 4 points in the thermodynamic cycle. *P*_r_ is the condenser heat rejection per unit time, which is also equal to the cooling power of the fan for the condenser (eq.14). *h_a_*(*v*_air_)is the overall heat transfer coefficient of a finned tube heat exchanger, which is the function of air velocity. The formula of *h_a_* is based on the empirical correlation of the Nusselt number of laminar flow on flat plates^13^ and the geometrical parameters of a common finned tube heat exchanger. *T*_airin_ and *T*_airout_ are the temperature for air flowing in and out of the condenser fins, respectively. *P*_com_ is the input power for the compressor. *η*_com_ is the compressor efficiency. *P*_fan_(*v*_air_) is the power consumption of the fan calculated by the fan affinity law. Hence, the COP of the traditional AC can be calculated by dividing *P*_load_ with *P*_total_.

$P_{\text{load}}\text{=}\frac{\text{d}m}{\text{d}t}(h_{1}-h_{4})$ (12)

$P_{\mathrm{r}}=\frac{\text{d}m}{\text{d}t}(h_{2}-h_{3})$ (13)

$P_{\mathrm{r}}=h_{a}(v_{air})\cdot(T_{\text{airout}}-T_{\text{airin}})$ (14)

$P_{\text{com}}=\frac{\text{d}m}{\text{d}t}(h_{2}-h_{1})/\eta_{\text{com}}$ (15)

$P_{\text{total}}=P_{\text{fan}}(v_{air})+P_{\text{com}}$ (16)

$\text{COP}=\frac{P_{\text{load}}}{P_{\text{total}}}$ (17)

Then we added radiatively-cooled water panel to the system to enhance the efficiency. For the cooling panels, thermodynamic equations (eq.18-eq.22) were given below. Note *p*_cool_ is cooling power density (W/m^2^) and *P*_cool_ is the cooling power (W). To account for the negative correlation between cooling power density and sub-ambient temperature drop (Δ*T*_cool_) due to the hemispherical ambient thermal radiance, the cooling power density is subtracted by 4.23*Δ*T*_cool_, which is based on Eriksson, T. S., and C. G. Granqvist’s research^14^. *S* is the effective roof area that could be utilized, which is assumed to be 60% of the model building rooftop area. *dm*_water_/d*t* is the water mass flow rate inside the tube. *C_water_* is the heat capacity of water. *h_w_*(*v*_water_) is the overall heat transfer coefficient of water approximated with the Dittus-Boelter equation for pipe flow. *T*_waterin_ and *T*_waterout_ are the temperature for water flowing in and out of the radiative cooling surface-plate heat exchanger, respectively. *P*_pump_(*v*_water_) is input water pump power, which is also calculated by the fan affinity law. The new COP could, therefore, be calculated by dividing *P*_load_ by *P*’_total_.

$(p_{\text{cool}}-4.23\cdot\Delta T_{\text{cool}})S=P_{\text{cool}}=\frac{\text{d}m_{\text{water}}}{\text{d}t}\text{C}_{\text{water}}\Delta T_{\text{cool}}$ (18)

$P'_{\mathrm{r}}=h_{a}(v_{\text{air}})\cdot(T_{\text{airout}}-T_{\text{airin}})\text{+}h_{w}(v_{\text{water}})\cdot\text{(}T_{\text{waterout}}-T_{\text{waterin}}\text{)}$ (19)

$P'_{\text{total}}=P_{\text{fan}}(v_{\text{air}})+P_{\text{pump}}(v_{\text{water}})+P_{\text{com}}(\Delta t_{\text{cool}})$ (20)

$\text{COP}_{\text{new}}=\frac{P_{\text{load}}}{P'_{\text{total}}}$ (21)

Finally, the cooling energy saved by using cooling materials can be demonstrated by:

$E_{saving,cool}=E\left(1-\frac{\text{COP}}{\text{COP}_{\text{new}}}\right)$ (22)

where *E* is the cooling electricity consumption with traditional AC, calculated by *EnergyPlus*

Heating-mode energy saving:

For heating energy saving, eq.23 and 24 were used to analyze the device performance. *P*_heating_ is the radiative heating power of the device. *I* is the global horizontal solar radiation obtained from TMY3 weather data^10^. *S* is the effective roof area that could be utilized, which is assumed to be 60% of the model building rooftop area. *α* is absorption coefficient of heating materials. *E*_saving,heat_ is the heating energy saving.

$P_{\text{heating}}=IS\alpha$ (23)

*E*_saving,heat_ = 3600**MIN* (*P_heating_*, *P_heating load_*) (24)

Through the above cooling model and heating model, the cooling and heating energy saving of each city per hour can be calculated. In dual-mode calculation, we can choose to operate in the mode that generates the maximum energy saving in that specific hour. That is, if the cooling saving is larger than heating saving, then we use cooling mode in this hour. Otherwise, heating mode would be taken into consideration.

*E*_saving,dual_ = *MAX* (*E*_saving,cool_, *E*_saving,heat_) (25)

Therefore, by arranging all cooling and heating energy saving in each hour of each city, we could get the annul energy saving in the U.S. in heating-only, cooling-only, and dual-mode approaches (see Fig. 5).

**Supplementary References**

[1] Hu, M. et al. Field test and preliminary analysis of a combined diurnal solar heating and nocturnal radiative cooling system. *Appl. Energy* **179**, 899-908 (2016).

[2] Vall, S. Medrano, M. Solé, C. & Castell, A. Combined radiative cooling and solar thermal collection: experimental proof of concept. *Energies* **13**, 893 (2020).

[3] Hu, M. et al. Performance assessment of a trifunctional system integrating solar PV, solar thermal, and radiative sky cooling. *Appl. Energy* **260**, 114167 (2020).

[4] Ono, M. Chen, K. Li, W. & Fan, S. Self-adaptive radiative cooling based on phase change materials. *Opt. express* **2**, A777-A787 (2018).

[5] Mandal, J. et al. Li_4_Ti_5_O_12_: A visible‐to‐infrared broadband electrochromic material for optical and thermal management. *Adv. Funct. Mater.* **28**, 1802180 (2018).

[6] Mandal, J. et al. Porous polymers with switchable optical transmittance for optical and thermal regulation. *Joule* **3**, 3088-3099 (2019).

[7] Zhao, et al. Switchable cavitation in silicone coatings for energy-saving cooling and heating. *Adv. Mater*. **32**, 2000870 (2020).

[8] Mandal, J. et al. Scalable, “Dip-and-Dry” Fabrication of a Wide-Angle Plasmonic Selective Absorber for High-Efficiency Solar–Thermal Energy Conversion. *Adv. Mater*. **29**, 1702156 (2017).

[9] Lord, S.D. A new software tool for computing Earth’s atmospheric transmission of near- and far-infrared radiation Available at: <https://ntrs.nasa.gov/search.jsp?R=19930010877>

[10] National Solar Radiation Data Base, 1991-2005 Update: Typical Meteorological Year 3; <https://rredc.nrel.gov/solar/old_data/nsrdb/1991-2005/tmy3/>. [Accessed May 28, 2020]

[11] Li et al. Radiative cooling resource maps for the contiguous United States. *J Renew Sustain Ener.* **11**, 036501 (2019).

[12] Fernandez, N., Wang, W., Alvine, K., Katipamula, S. Energy savings potential of radiative cooling technologies. PNNL-24904, (2015).

[13] (Frank P.; DeWitt, David P. (2007). Fundamentals of heat and mass transfer (6th ed.). Hoboken: Wiley.)

[14] Eriksson, T. S., and C. G. Granqvist. "Radiative cooling computed for model atmospheres." *Appl. Optics* **21**, 4381-4388 (1982).
