# Supplementary material for: Integration of daytime radiative cooling and solar heating for year-round energy saving in buildings
Source: Nat Commun. 2020 Nov 30;11:6101. doi: 10.1038/s41467-020-19790-x (PMC7705009; doi:10.1038/s41467-020-19790-x)
Supplement: Supplementary file 3 — Description of Additional Supplementary Files [file 41467_2020_19790_MOESM3_ESM.pdf]

Description of Additional Supplementary Files

File Name: Supplementary Movie 1
Description: Movie of switching process between heat mode and cool mode of the dual-mode device.
